# Inhibition of plasmin-mediated TAFI activation may affect development but not progression of abdominal aortic aneurysms

**DOI:** 10.1371/journal.pone.0177117

**Published:** 2017-05-04

**Authors:** Katherine Bridge, Charlotte Revill, Fraser Macrae, Marc Bailey, Nadira Yuldasheva, Stephen Wheatcroft, Roger Butlin, Richard Foster, D. Julian Scott, Ann Gils, Robert Ariёns

**Affiliations:** 1 Thrombosis and Tissue Repair Group, Division of Cardiovascular and Diabetes Research, Leeds institute for Cardiovascular and Metabolic Research, University of Leeds, Leeds, United Kingdom; 2 The Leeds Vascular Institute, Leeds General Infirmary, Leeds, United Kingdom; 3 School of Chemistry, University of Leeds, Leeds, United Kingdom; 4 KU Leuven- University of Leuven, Department of Pharmaceutical and Pharmacological Sciences, Laboratory for Therapeutic and Diagnostic Antibodies, Leuven, Belgium; Monash University, AUSTRALIA

## Abstract

**Objective:**

Thrombin-activatable fibrinolysis inhibitor (TAFI) reduces the breakdown of fibrin clots through its action as an indirect inhibitor of plasmin. Studies in TAFI-deficient mice have implicated a potential role for TAFI in Abdominal Aortic Aneurysm (AAA) disease. The role of TAFI inhibition on AAA formation in adult ApoE-/- mice is unknown. The aim of this paper was to investigate the effects of TAFI inhibition on AAA development and progression.

**Methods:**

Using the Angiotensin II model of AAA, male ApoE-/- mice were infused with Angiotensin II 750ng/kg/min with or without a monoclonal antibody inhibitor of plasmin-mediated activation of TAFI, MA-TCK26D6, or a competitive small molecule inhibitor of TAFI, UK-396082.

**Results:**

Inhibition of TAFI in the Angiotensin II model resulted in a decrease in the mortality associated with AAA rupture (from 40.0% to 16.6% with MA-TCK26D6 (log-rank Mantel Cox test p = 0.16), and 8.3% with UK-396082 (log-rank Mantel Cox test p = 0.05)). Inhibition of plasmin-mediated TAFI activation reduced the incidence of AAA from 52.4% to 30.0%. However, late treatment with MA-TCK26D6 once AAA were already established had no effect on the progression of AAA in this model.

**Conclusions:**

The formation of intra-mural thrombus is responsible for the dissection and early rupture in the angiotensin II model of AAA, and this process can be prevented through inhibition of TAFI. Late treatment with a TAFI inhibitor does not prevent AAA progression. These data may indicate a role for inhibition of plasmin-mediated TAFI activation in the early stages of AAA development, but not in its progression.

## Introduction

An Abdominal Aortic Aneurysm (AAA) is a permanent, focal dilatation of the descending abdominal aorta. It most commonly occurs in men over the age of 65 years [1]. The natural history of an AAA is expansion with eventual rupture, and, despite an apparent global decrease in rupture rate [2], ruptured AAA is still responsible for over 8000 deaths per annum in the USA [3]. Large AAA are characteristically accompanied by the presence of an intra-luminal thrombus (ILT) [4]. The ILT is an independent risk factor for expansion and rupture of AAA, and, through the action of plasmin- and metalloproteinase-mediated proteolysis, is thought to directly contribute to the breakdown of the underlying aortic wall [5]. Even beyond the ILT, there is evidence of systemic changes in clotting in patients with AAA. In line with a number of cardiovascular disease states, including myocardial infarction, stroke and peripheral arterial disease [6], patients with AAA develop denser *ex-vivo* clots which are more resistant to lysis [7]. The exact mechanism for this change, and whether this represents cause or effect of underlying cardiovascular disease states, remains to be elucidated. There is evidence for a generalized increase in fibrinolytic activity in this group of patients, with elevated plasma levels of plasmin-antiplasmin complexes (PAP) [8], D-dimer, thrombin-antithrombin (TAT) and prothrombin fragments F1+2 [9]. Occlusion of the aneurysm sac, as occurs through endovascular repair, does not result in the reduction of these parameters back to normal levels [10], implying that there is an ongoing pathological phenotype in this patient group which occurs beyond the simple presence of an AAA. What is known, however, is that patients with AAA are frequently affected by other atherothombotic cardiovascular diseases, in particular coronary artery disease, and independent of all other risk factors remain at an increased risk of cardiovascular death [11].

Thrombin-activatable fibrinolysis inhibitor (TAFI) is a physiological inhibitor of plasmin-mediated fibrinolysis. By cleaving C-terminal lysine residues from partially degraded fibrin molecules, activated TAFI (TAFIa) prevents the co-localisation of plasminogen and tPA onto the surface of the fibrin clot, thereby reducing the production of plasmin, and thus inhibiting fibrin clot breakdown [12]. TAFIa also has anti-inflammatory properties, and through its cleavage of C3a, C5a, thrombin-cleaved osteopontin (OPN) and bradykinin, acts to counteract some of the inflammatory sequelae of thrombin activation *in-vivo* [13]. TAFI has become a popular target for new anti-thrombotic agents, with a series of antibodies, nanobodies and small molecule inhibitors being developed against TAFI [14–16]. These include monoclonal antibody inhibitors such as MA-TCK26D6, which specifically inhibits plasmin-mediated activation of TAFI, and has been shown to reduce thromboembolism *in-vivo* in a murine model [17], and, in a diabody confirmation with a plasminogen-activator inhibitor-1 (PAI-1) antibody, effectively reduced lesion size and improved functional outcomes in a stroke model [18]. Evidence for a potential role for TAFI in AAA has largely been inferred from a single study in TAFI knockout mice, which developed larger aneurysms that were more prone to ruptures, upon porcine pancreatic elastase (PPE) infusion compared with wild type controls [19]. There are only two previous studies of TAFI in humans with AAA. The conclusions of both of these studies were based on very small populations, but demonstrated an apparent increase in TAFI activity in patients with AAA compared with control subjects [20, 21]. Due to the implication of TAFI as a potentially important molecule in AAA disease, the aim of this paper was to investigate the role of inhibition of TAFI in AAA development and progression in adult Apolipoprotein E deficient (ApoE-/-) mice. This was achieved using a monoclonal antibody (MA-TCK26D6), which impairs the activation of TAFI mediated by plasmin, specifically preventing the interaction between TAFIa and fibrin, but not affecting its binding to small molecules such as OPN, C3a and C5a, and a competitive small molecule inhibitor of active TAFI, UK-396082, which binds directly to the active catalytic site of TAFI, and thus prevents all of its action (anti-fibrinolytic and anti-inflammatory) [22].

## Materials and methods

### AAA formation in the Angiotensin II model of AAA

All animal studies were carried out under the regulations laid out by the UK Home Office under project licence 40/3523, and were approved by local ethics committees at the University of Leeds. Animals were housed in groups of up to 5 in an individually ventilated cage system within a purpose built animal unit. They had free access to chow diet and water for the duration of the experiment, and were maintained in a 12 hour light-dark cycle. ApoE-/- mice for use in the study were bred in house from an established colony at the University of Leeds. AAA were induced using the Angiotensin II model developed by Daugherty et al [23]. In brief, male, ApoE-/- mice aged between 12–14 weeks, which were backcrossed for ten generations on a C57Bl/6J background, were implanted with Alzet^®^ osmotic mini-pumps delivering 750ng/kg/min Angiotensin II, Ang II, (n = 35) or Saline control (n = 23) under general anaesthesia induced with inhaled isoflurane. Each pump continuously delivered treatment for 28 days. Before recovery from anaesthesia, all mice were administered with a single dose of Buprenorphine (0.5mg/kg) via intra-peritoneal injection. Once awake, mice were placed in a warm, clean cage, and returned to their normal cage environment once fully recovered. Mice were monitored at least every 24 hours throughout the 28 day experiment. If any animal showed any signs of distress prior to the end point of the experiment (such as hunched posture, partially closed eyes, changes to respiration, rough hair, withdrawal from the group), they were sacrificed using schedule 1 approved methods.

### Non-invasive monitoring of heart rate and blood pressure

In the second week following mini-pump insertion, mice underwent non-invasive monitoring of their heart rate (HR) and blood pressure (BP) using the CODA device (Kent Scientific Corporation).

### Inhibition of TAFI in the Angiotensin II model of AAA

Once the model had been established, TAFI inhibitors (MA-TCK26D6 and UK-396082, n = 12 in each group) were given in combination with Ang II under the same general anaesthetic and compared with mice receiving Ang II alone. MA-TCK26D6, or saline control, was administered as a single intravenous injection of 436 μg per mouse (equivalent to 17 mg/kg for an average 25 g mouse) under direct vision via the femoral vein under general anaesthesia. UK-396082 was administered at a dose of 30 mg/kg/min via a second subcutaneous Alzet^®^ 1004 mini-osmotic pump, which was inserted as described above. In a subsequent group, (n = 14), due to a positive response in the prevention of development of AAA, intravenous MA-TCK26D6 (436μg) was administered one week after the initiation of the Ang II infusion, and compared with mice treated with a saline control injection at one week (n = 9).

### Ultrasound scanning (USS) of the abdominal aorta

AAA progression was monitored using the Vevo^®^2100 pre-clinical ultrasound scanner, with VevoVasc software used to measure aortic volume and distensibility. For the Vevo^®^2100 USS imaging, mice were anaesthetised using volatile isoflurane. A MS550D MicroScan^™^ transducer at 22–55 MHz was used for collection of the scan images, with a focus depth of 4 mm. A series of images of the abdominal aorta were taken, both in cross section and longitudinally. Colour Doppler and pulse wave Doppler were used to ensure the positioning of the abdominal aorta. A 3D USS image of the suprarenal aorta was obtained for the 10.5 mm superior to the right renal artery, in the position where any AAA would be expected to be located. An electrocardiogram-gated (ECG-gated) image was obtained of the longitudinal aorta in order to assess distensibility. Following the scan, the mice were recovered in a warm clean cage, before being returned to their usual maintenance environment.

Scan images were processed remotely using VevoLab 1.7.0 and VevoVasc software. The aortic diameter was measured in cross section at a position 5 mm proximal to the right renal artery. Longitudinally, the aortic diameter was measured in its maximal diameter using an ECG-gated image at maximal systole. Distensibility was calculated using an ECG-gated image and VevoVasc software, and was a measurement of how much the aortic wall moved from the diastolic position with each cardiac cycle. The aortic volume was calculated using a 3D reconstruction of the 10.5 mm of the abdominal aorta proximal to the right renal artery, which is the site of any AAA formation.

### Blood collection and harvesting of the aorta

At 4 weeks post-pump implantation, mice were anaesthetised using inhaled isoflurane at 5 litres/min, then maintained at 2 litres/minute until the point of death. Whole blood was collected from the inferior vena cava (IVC), onto 0.1 mol/L sodium citrate at a ratio of 9 parts blood to 1 part citrate. Following exsanguination, the left ventricle was cannulated, and the circulation perfused with 8–10 mls of phosphate buffered saline (PBS). This PBS infusion was then followed by 5 mls of 4% paraformaldehyde, PFA (P6148, Sigma-Aldrich Co. LLC.). Macroscopic imaging of the aorta was undertaken using the OPMI-PICO video micrometer (Carl Zeiss AG).

Samples of whole blood collected from the IVC were kept on ice until processing. They were then centrifuged at 12, 000 rcf for 20 minutes using a table-top micro-centrifuge to yield platelet poor plasma, flash frozen in liquid nitrogen, and stored at -40°C until analysis.

The size of the aorta was measured in-situ, and the aortic ratio calculated (being the ratio of the abdominal aorta/the normal thoracic aorta, with a normal value of 1). The aorta was then harvested en-bloc from the mid-thoracic level to below the left renal artery. In preparation for histological examination, the aorta was placed in 4% PFA for a minimum of 24 hours before processing. Samples were dehydrated on a Leica TP1020 tissue processing carousel and embedded in paraffin. Blocks were sectioned using a Leica RM2125 microtome into 5 μm sections, and collected onto slides (4951PLUS4 Superfrost^™^Plus, Thermo Scientific Ltd), left to dry at 37°C overnight, and stored before staining. Slides were stained for elastin using a millers elastin stain and fibrin using martius scarlet blue. Slides were imaged using an Olympus BX40 Dual View Microscope and Image Pro-Plus 8.0 at 4-20x magnifications.

### Turbidity and turbidity-lysis

The turbidity and turbidity-lysis protocols which have previously been used in patient samples [7] were adapted for use in murine plasma. Murine plasma samples (100μl, diluted 1:4 in 0.05mol/L HEPES buffer with 0.1% BSA, pH 7.4) were added to each well of a 96-well Greiner bio-one microtitre plate. An activation mix (25μl) containing murine thrombin (MCT-5020-50, Cambridge Biosciences, UK), calcium chloride (CaCl_2_), with or without human recombinant tissue plasmingen activator (tPA) in a 0.05mol/L HEPES buffer, were added to each well. Final concentrations per well were 0.2units/ml thrombin, 0.01mol/L CaCl_2_ and 0.7pmol/L tPA. Plasma samples were run in duplicate both with and without the addition of tPA. The absorbance was measured every 12 seconds for 90 minutes at 405nm.

### Confocal microscopy

For each plasma sample, a clot containing murine fluorescein isothiocyanate-labelled (FITC-labelled) fibrinogen was prepared. A mixture containing 30μl of plasma, CaCl_2_ (final concentration 0.02mol/L) and murine FITC-fibrinogen (Oxford Biomedical Research, USA, final concentration 50μg/ml) was made in TBS up to a total volume of 54μl. A reaction mix (6 μl) containing murine thrombin (MCT-5020-50, Cambridge Biosciences, UK), final concentration 0.5 units/ml, was added to the samples. 30μl of this mixture was immediately transferred into one side of a capillary confocal slide (80601 Thistle Scientific Ltd. UK), and the sample monitored to ensure it crossed onto the other side of the slide by capillary action. Once prepared, the slides were kept in a dark humidity chamber at room temperature for 2–4 hours in order to ensure that the clot was completely formed. Slides were imaged using a Zeiss LSM-700 inverted confocal microscope at 63x magnification. Images were obtained from at least 4 different parts of the clot. Images were analysed using Image J.

### Statistical analysis

Raw data obtained in the laboratory were initially collected into Microsoft Excel. GraphPad Prism v6 was used to represent the data graphically. Statistical tests were performed using IBM SPSS Statistics v20 (SPSS Inc. Chicago, Illinois, USA) and GraphPad Prism v6. Statistically significant results were taken as p<0.05.

All animal work was carried out within UK Home Office Regulations, under project licence number 40/3523, and approved by the local ethics committee. The sample size for the study was based on pilot data relating to the Ang II model in our institution, with Ang II given at a dose of 750 ng/kg/min. Using this data, in order to detect an increase in diameter of 1.3x (reverting from the mean for AAA in our pilot data 2.3±0.9 to the normal 1.0±0.2), with 90% power at p<0.05, 6 animals would be needed to appreciate an effect. Given the known mortality of this model of up to 40% 12 mice were used per group. Data was analysed using Student T-test, and presented as mean±standard deviation. Differences in survival rates were analysed using Log-rank Mantel Cox tests.

## Results

**Fig 1:**
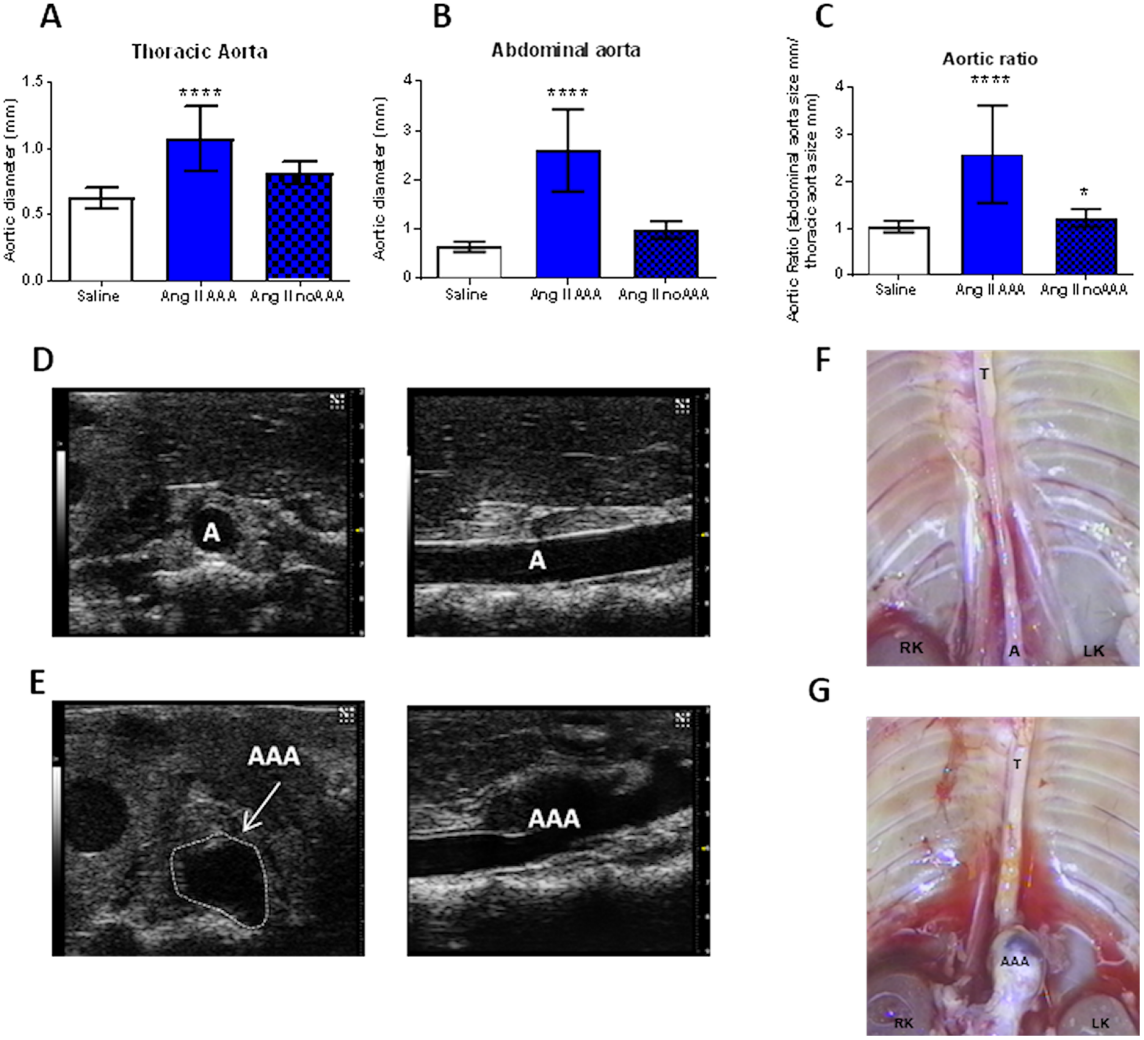
Characterisation of the Angiotensin II model of AAA. Apo E-/- mice were treated with Angiotensin II 750ng/kg/min or NaCl 0.9% via subcutaneous mini-pump for 28 days. In mice surviving to 28 days (Ang II n = 21 and NaCl 0.9% n = 23) images were taken of the aorta, and measurements taken to determine the size of both the thoracic aorta and the supra-renal abdominal aorta. Mice were classified as AAA (n = 11) or no AAA (n = 10), based upon the presence of an AAA (defined as aortic ratio >1.5). Panel A There was an increase in the size of the thoracic aorta in all mice treated with Ang II. Panel B The size of the abdominal aorta also increased in all mice treated with Ang II, with a more dramatic increase in those with a macroscopic AAA. Panel C The aortic ratio (abdominal/thoracic aorta) in mice developing AAA. Panels D—E AAA growth in this model was monitored using pre-clinical ultra sound scanning (Vevo 2100). At baseline, the aorta was uniform in shape, with smooth walls (Panel D). After as little as 7 days, large AAA were evident, and, after 21 days, established AAA, with irregular, dilated lumen, could be visualised (Panel E). A—aorta, AAA—abdominal aortic aneurysm, white dotted line demonstrates the maximal diameter of the AAA. Panel F Representative image of control aorta at 28 days Panel G Representative image of Angiotensin II induced AAA at 28 days T—thoracic aorta, A—abdominal aorta, RK—right kidney, LK—left kidney, AAA—abdominal aortic aneurysm. Data is shown as mean±standard deviation, * p<0.05 compared to control by student t-test, **** p<0.0001 compared to control by student t-test.

ApoE-/- mice were treated with Ang II 750 ng/kg/min or NaCl 0.9% via subcutaneous mini-pump for 28 days. In mice surviving to 28 days (Ang II n = 21 and NaCl 0.9% n = 23) images were taken of the aorta, and measurements taken to determine the size of both the thoracic aorta and the supra-renal abdominal aorta. Mice were classified as AAA (n = 11) or no AAA (n = 10), based on the aortic measurements, with AAA defined as abdominal/thoracic aortic ratio >1.5. There was an increase in the size of the thoracic aorta in all mice treated with Ang II; NaCl 0.9% 0.62 ± 0.08 mm, Ang II no AAA 0.81 ± 0.09 mm, Ang II AAA 1.07 ± 0.25mm (p<0.0001). The size of the abdominal aorta also increased in all mice treated with Ang II, with a more dramatic increase in those with a macroscopic AAA; NaCl 0.9% 0.9% 0.65 ± 0.10 mm, Ang II no AAA 0.99 ± 0.18 mm, Ang II AAA 2.60 ± 0.83 mm (p<0.0001). The aortic ratio in mice developing AAA was 2.59 ± 1.04, whilst in control mice treated with saline it was 1.04 ± 0.12 (p<0.0001; See Fig 1A, 1B and 1C). There was no change in the fibrin clot structure in mice developing AAA compared with controls (see S1 Fig). These data suggest that the effects of Ang II on AAA formation are mediated through vascular effects that do not include effects on fibrin clot structure at this stage of AAA development.

**Fig 2:**
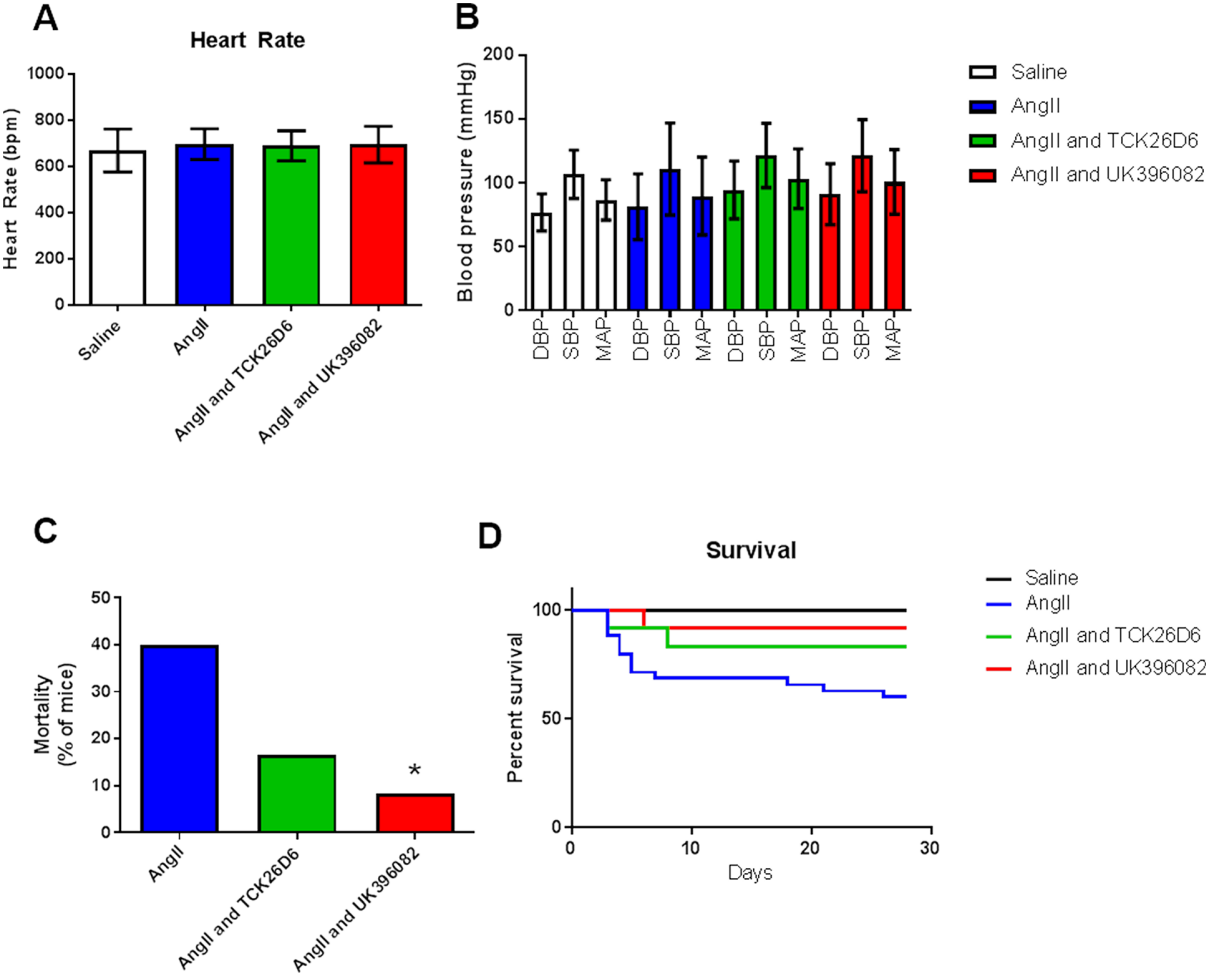
The effect of TAFI inhibition on mortality in the Angiotensin II model of AAA. Panel A and B Blood pressure (BP) and heart rate (HR) measurements taken in all groups of mice in the second week of the experimental period using the CODA non-invasive BP device. Panel C and D Mortality of mice treated with Ang II, compared with NaCl 0.9% or Ang II plus MA-TCK26D6 or UK-396082. Data is shown as mean±standard deviation. Mortality in this model of AAA typically occurred early (Days 3–8 post initiation of Ang II infusion). * p<0.05 compared to Angiotensin II alone by Chi-Squared testing.

There was no change in the blood pressure (BP) or heart rates (HR) of mice treated with Ang II alone, or in combination with either of the two inhibitors. HRs were within normal limits in all four groups: Saline 670.8 ± 92.5 bpm, Ang II 697.4 ± 66.19bpm, Ang II and MA-TCK26D6 690.6 ± 65.19bpm, Ang II and UK-396082 695.8 ± 78.41bpm (Fig 2A). BP readings were also within normal limits for all four groups: Saline 107/77, Ang II 111/81, Ang II and MA-TCK26D6 121/94 and Ang II and UK-396082 121/91 (Fig 2B).

Treatment with either of the two TAFI inhibitors (MA-TCK26D6 or UK-396082) in combination with Ang II resulted in a decrease in the mortality usually associated with this model. The mortality of mice treated with Ang II 750 ng/kg/min via a subcutaneous mini pump (n = 35) was 40.0%. Mortality in the NaCl 0.9% control group (n = 23) was 0%. When MA-TCK26D6 was given in combination with Ang II (n = 12), mortality rates fell to 16.6% (log-rank mantel cox test p = 0.16, Fig 2C and 2D). When UK-396082 was delivered in combination with Ang II (n = 12), mortality rates fell to 8.3% (log-rank mantel cox test p = 0.05; Fig 2C and 2D).

**Fig 3:**
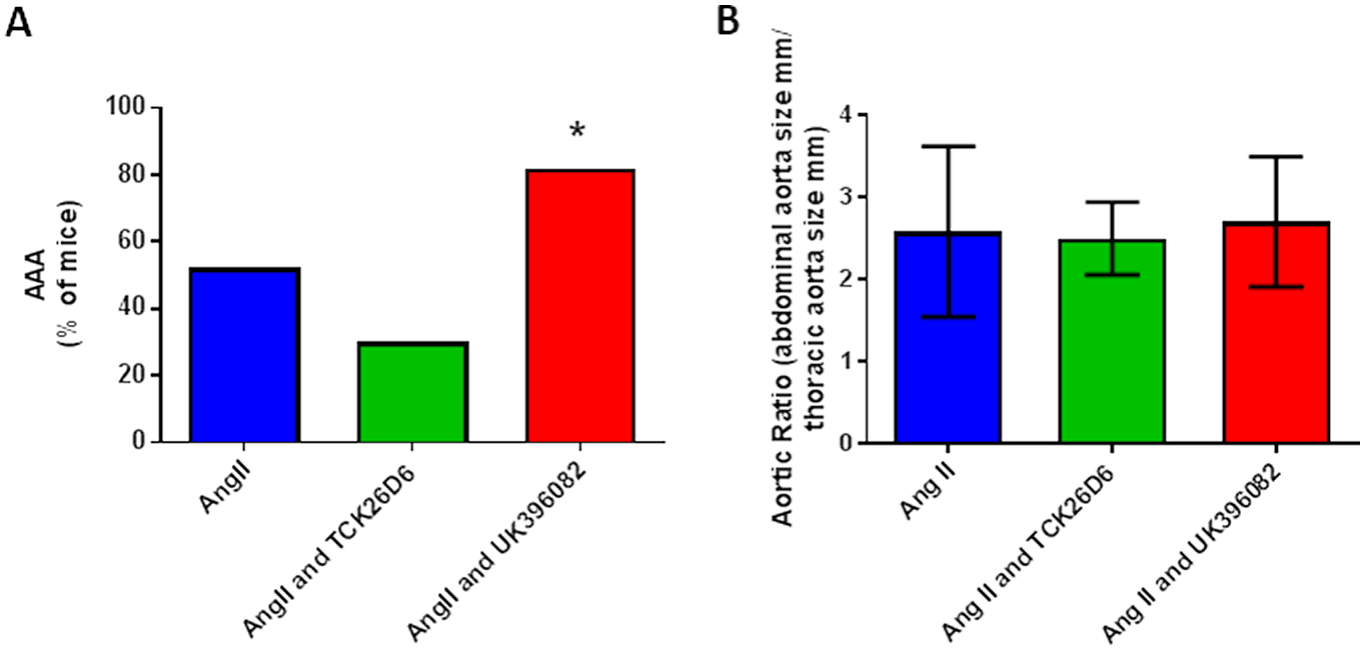
AAA formation is altered in the presence of a TAFI-inhibitor. Mice were treated with saline control, NaCl 0.9%, (n = 23), Ang II (n = 35), Ang II and MA-TCK26D6 (n = 12) or Ang II and UK-396082 (n = 12) for 28 days. Panel A Incidence of AAA in each group. Panel B AAA size. Data is shown as mean±standard deviation. * p<0.05 compared to Angiotensin II alone by Chi-Squared testing.

**Fig 4:**
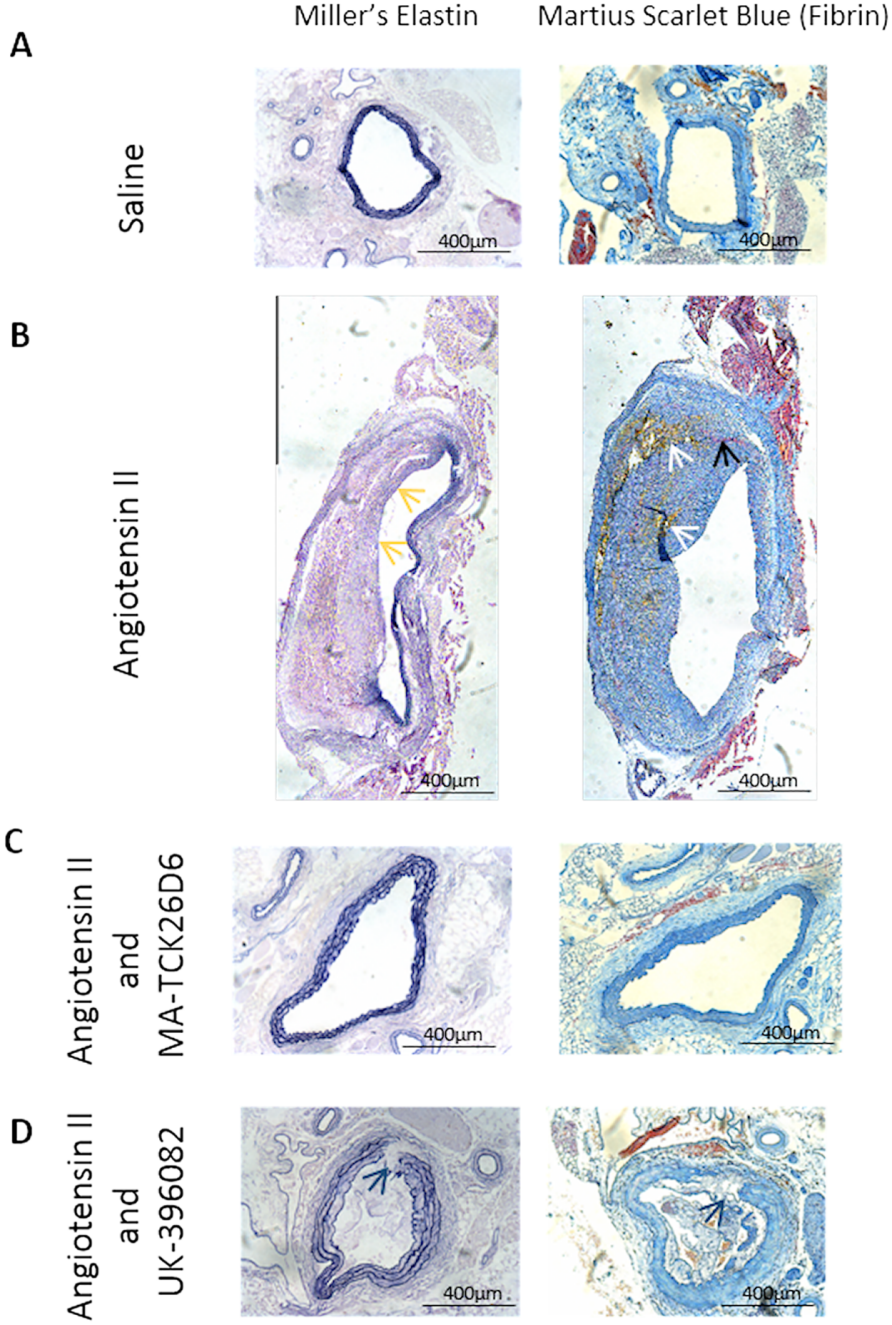
Histological examination of the abdominal aorta in the Angiotensin II model of AAA following treatment with a TAFI inhibitor. Supra-renal aortas of mice infused with Saline control, Ang II, Ang II and MA-TCK26D6 or Ang II and UK-396082 for 28 days were fixed and stained for elastin, collagen and fibrin, and imaged using an Olympus BX40 Dual View Microscope. Row A shows images from a mouse treated with NaCl 0.9% (control). In mice treated with Ang II (Row B), changes characteristic of AAA are demonstrated, with loss of elastin from within the structural matrix of the aorta (yellow arrow), and evidence of intra-mural thrombus, with erythrocyte deposition (white arrow) and fibrin formation (black arrow) present in the expanded medial layer. Row C shows images of aortas taken from a mouse treated with Angiotensin II and MA-TCK26D6. In this group, only 30% of mice surviving to 28 days developed AAA, and the elastin layer remains intact, with no thrombus seen within the aortic wall. In mice treated with Ang II 750 ng/kg/min in combination with UK-396082 30mg/kg/min, over 80% of mice had developed AAA at 28 days. There was gross heterogeneity in the macroscopic and microscopic appearance of these AAA. Samples from some mice even displayed evidence of complete elastin breaks/dissections through the internal elastic lamina (blue arrow). (Row D).

Although there was a reduction in the rate of mortality with both of the TAFI inhibitors, the rate of AAA formation differed dependent on the inhibitor used. With MA-TCK26D6, there was a reduction in the incidence of AAA in mice surviving to 28 days (reduced to 30.0% from 52.4%). With UK-396082, the incidence of AAA in mice surviving to 28 days was increased to 81.9% from 52.4% (p = 0.03). In fact, when the presence of an AAA at 28 days was taken together with mortality due to rupture, the incidence of AAA was almost equivalent between the Ang II and Ang II with UK-396082 groups (71% vs. 83%), compared with the Ang II and MA-TCK26D6 group, where the combined response as measured by rupture or presence of an AAA was only 42% (Fig 3A). When AAA did occur, there was no significant difference in the size of AAA in the different groups (Aortic ratio Ang II 2.6 ± 1.0 vs. Ang II and MA-TCK26D6 2.5 ± 0.4 vs. Ang II and UK-396082 2.7 ± 0.8; Fig 3B). Histological examination of the aortas taken from mice treated with the TAFI inhibitors, however, showed differences between the two inhibitor groups and the mice receiving only Ang II, even when macroscopic AAA did occur. In mice treated with Ang II, there was loss of elastin from within the structural matrix of the aortic wall, and evidence of intra-mural thrombus, with erythrocyte deposition and fibrin formation present in the expanded medial layer (Fig 4B). In mice receiving both Ang II and MA-TCK26D6 that did develop AAA, there was no evidence of intra-luminal thrombus, but there was evidence of deregulated elastin and collagen within the internal elastic lamina and media (Fig 4C). In mice treated with Ang II in combination with UK-396082, over 80% of mice had developed AAA at 28 days. There was gross heterogeneity in the macroscopic and microscopic appearance of these AAA. Unlike in the aortas from mice treated with Ang II alone, there was evidence of intra-luminal thrombus in a number of sections taken from mice treated with UK-396082. Some mice displayed evidence of complete elastin breaks/dissections through the internal elastic lamina, whilst others showed elastin thinning and gross aortic dilatation without an area of specific elastin breakage (Fig 4D).

**Fig 5:**
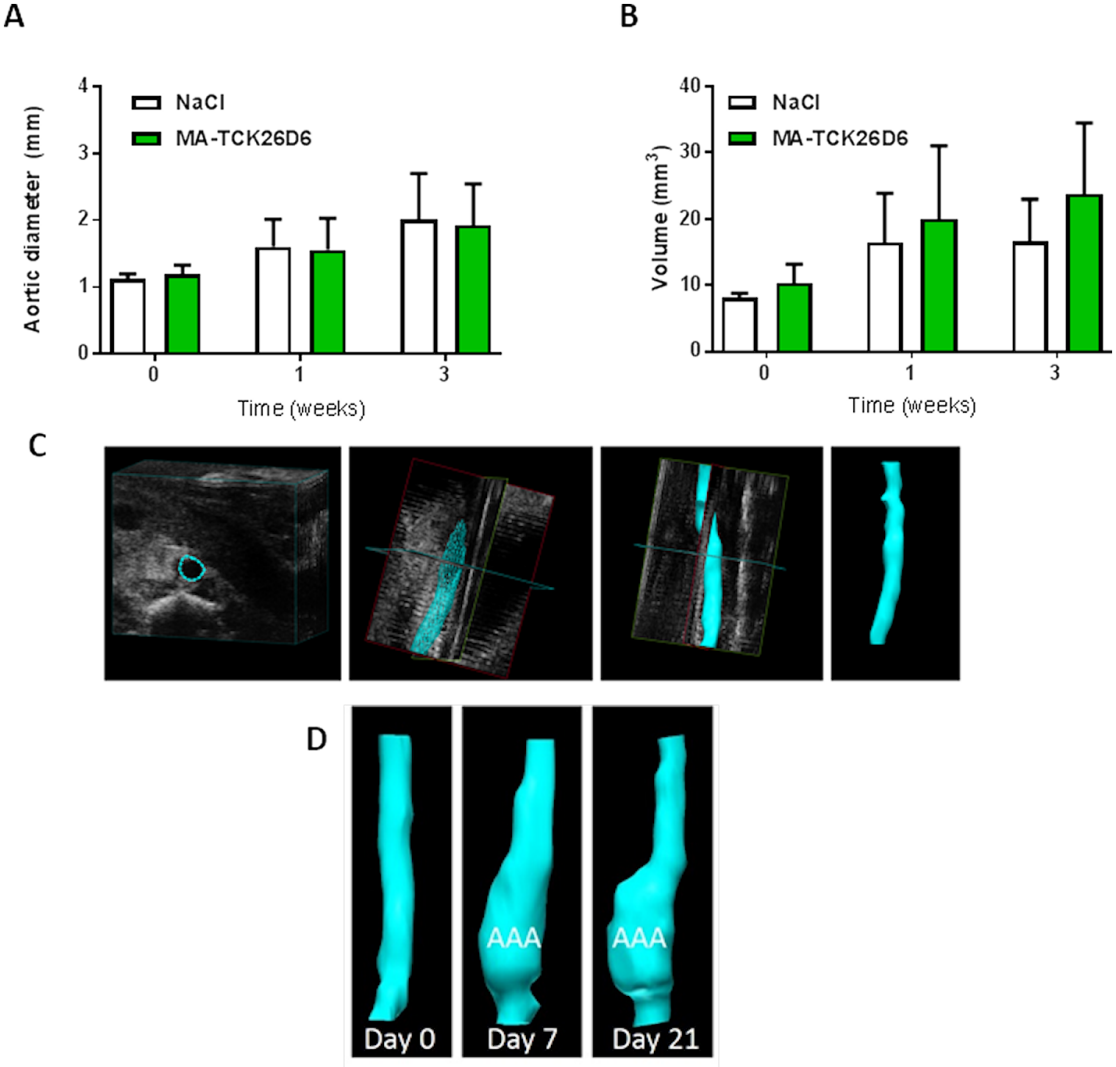
There is no effect of TAFI inhibition of the growth of an established AAA in the Angiotensin II model. AAA were induced in hyperlipidaemic mice by infusion of Ang II 750 ng/kg/min. After 1 week, mice were treated with a single injection of either MA-TCK26D6 or NaCl control. AAA progression in both groups was evaluated at 2 weeks post injection using Vevo2100 pre-clinical ultrasound scanning, and 3D reconstructions of the aortic segment at risk of AAA formation were created. Panel A Aortic diameter, Panel B Aortic volume, Panel C The process of creating the 3D aortic reconstruction. Panel D Example 3D reconstruction showing AAA progression over time (0, 1 and 3 weeks post Angiotensin II infusion). Data is shown as mean±standard deviation.

Delayed treatment with MA-TCK26D6 one week after Ang II infusion (once an AAA was established) did not affect the progression of AAA in this model. After two weeks, there was no difference in the cross-sectional diameter of AAA in mice receiving the treatment versus those given a control injection (MA-TCK26D6 1.93±0.62mm vs. NaCl 2.02±0.69 mm, Fig 5A). In order to better quantify the heterogeneous expansion of the aorta, the volume of the 10.5 mm of aorta proximal to the right renal artery was reconstructed. The increase in volume over time was also comparable between the two groups, see Fig 5B.

**Fig 6:**
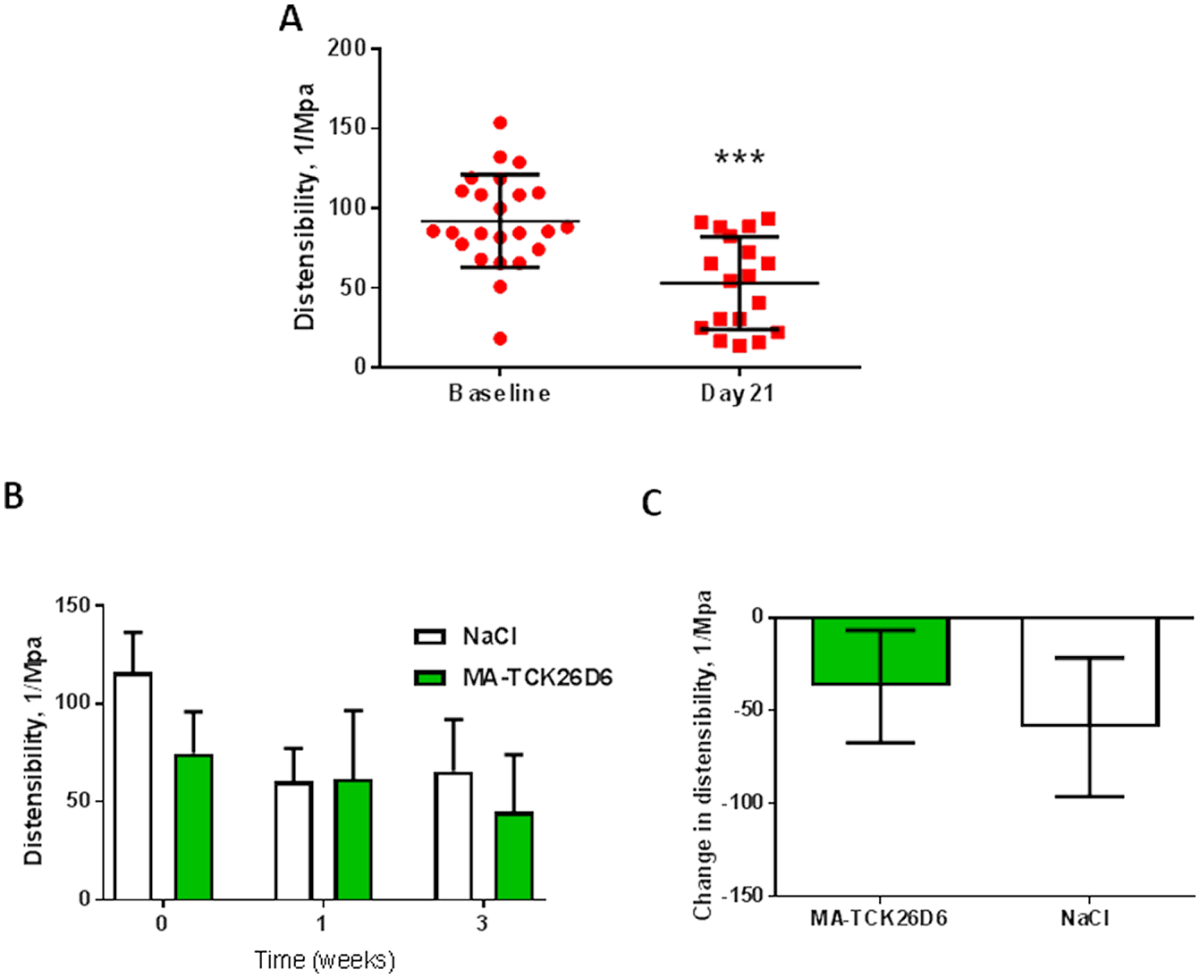
Aortic distensibility decreases with AAA formation, but is not affected by TAFI-inhibition, in the Angiotensin II model of AAA. The aorta was imaged in longitudinal section using the Vevo2100 scanner, and the distensibility measured using ECG-gated images and VevoVasc software. Panel A shows aortic wall distensibility in all mice with AAA (MA-TCK26D6 treated and sham treated). One week following initiation of Ang II infusion, mice received either MA-TCK26D6 or control (NaCl 0.9%) as an IV injection via the femoral vein. Panel B and C There was no difference in the change of distensibility by week 3 in mice receiving treatment with MA-TCK26D6 at 1 week compared with controls (data shown is change in distensibility between week 1 and week 3). Data is shown as mean±standard deviation, *** p = 0.001 compared to baseline by student t-test.

Using an ECG-gated ultrasound image of the abdominal aorta (taken in longitudinal section), the distensibility of the aortic wall was measured using VevoVasc software. The overall distensibility across both groups decreased as AAA developed (Baseline 91.92 ± 29.08 1/Mpa falling to 53.11 ± 29.03 1/Mpa, p = 0.0001, Fig 6A). Although there was some heterogeneity between animals, there was no difference in the change of distensibility in those mice receiving treatment with MA-TCK26D6 compared with controls (MA-TCK26D6 decreased by 37.01 ± 30.19 1/Mpa vs. NaCl decreased by 58.88 ± 37.31 1/Mpa), see Fig 6B and 6C.

## Discussion

Our study has demonstrated a role for TAFI in the early stages of AAA development in the Ang II model of AAA. By using two inhibitors that regulate different aspects of TAFI function, our data indicate the likely relative importance of inhibition of plasmin-mediated TAFI activation compared with total TAFI inhibition. For both inhibitors, mortality was reduced in the Ang II model of AAA. However, it was only the targeted inhibition of plasmin-mediated TAFI activation that appeared to confer a benefit in terms of a reduction in the incidence of AAA. Our data also indicate that the role of plasmin-mediated TAFI activation is confined to the early stages of the model, as late treatment with the same inhibitor inferred no benefit in terms of AAA progression.

The only previous *in-vivo* study of TAFI and AAA was performed in TAFI-/- mice using the porcine pancreatic elastase infusion model, with an increase in the incidence of AAA and the rupture rate in mice deficient in TAFI [19]. There are advantages and disadvantages to different animal models for AAA. The Ang II model was used for this study as it better replicates the thrombosis and fibrinolysis aspects of the AAA disease process, that is, it results in the production of a thrombus with a subsequent inflammatory response [23, 24]. We showed that the mortality associated with this model was decreased when MA-TCK26D6 was delivered at the initiation of the Ang II infusion, and this effect was also demonstrated using an inhibitor of all active TAFI, UK-396082. This implicates the importance of thrombus formation in this model. The initial stage of AAA development in this model involves dissection of the intima, followed by the development of an intra-mural thrombus. For both MA-TCK26D6 and UK-396082, the inhibition of TAFIa will lead to a reduction in the deposition and/or an increase in the resolution of the intra-mural thrombus that develops following aortic dissection. In support of this, there was no evidence of intra-mural thrombus on histology after 28 days. As mortality in this model classically occurs within the first 7 days after initiation of the Ang II infusion, we hypothesise that the formation of intra-mural thrombus early in the Ang II model of AAA increases the incidence of complete aortic dissection, and does not provide, as may be expected, a protective mechanism against aortic rupture.

Although inhibition of TAFI via either route resulted in a survival advantage in this model, the incidence of AAA was affected in different ways dependent on the route of inhibition. When UK-396082 was administered in conjunction with Ang II, there was an increase in the incidence of AAA at 28 days, however, once the reduced rate of rupture in this group was taken into account, there was no overall change in Ang II response, a result that is more in keeping with the previous study in the TAFI-/- mouse [19]. However, when plasmin-mediated activation of TAFI was inhibited using MA-TCK26D6, there was a reduction in the incidence of AAA, as indicated by both a reduction in rupture and also a reduction in the presence of an AAA at 28 days. MA-TCK26D6, whilst inhibiting the effects of TAFIa on fibrinolysis, preserves all of TAFIa’s anti-inflammatory properties. This leads to the hypothesis that once an intra-mural thrombus has formed, the driver for the ongoing development of AAA may be more related to inflammatory processes rather than ongoing thrombosis. When no inhibitor is used, there is evidence of intra-mural thrombus formation, and then a subsequent inflammatory response, which drives the breakdown of elastin within the vessel wall and ultimately results in AAA formation. When the UK-396082 inhibitor was used, the initial intra-mural thrombus would not have been as stable or long lasting as in the control. However, due to the inhibition of TAFIa’s anti-inflammatory properties in addition to its anti-fibrinolytic properties, we hypothesise that in this case, there would be unregulated inflammation at the site of the initial insult. Even in the absence of a thrombus to drive an ongoing proteolytic reaction, this unregulated inflammation would be sufficient to destroy the elastin of the vessel wall, and thus lead to AAA formation. The opposite was seen in mice receiving MA-TCK26D6, the inhibitor of plasmin-mediated TAFI activation. In this instance, again, the inhibition of TAFI would have prevented the formation of an intra-mural thrombus. However, in these mice, the anti-inflammatory properties of TAFI against small molecule inhibitors such as C3a, C5a and OPN would have remained intact, and there should therefore have been normal regulation of any inflammatory response by TAFIa. We hypothesise that in this case, the rapid resolution of any intra-mural thrombus and the relatively reduced inflammatory response (in comparison to UK-396082), result in resolution of the initial defect without subsequent aneurysm formation. This contrasts with the conclusion of the only previous *in-vivo* study. These authors showed that in their model, the administration of PPE into OPN-/- mice did not result in enhanced AAA formation, implying that it was not an unregulated inflammatory response that resulted in AAA formation in the TAFI-/- mice. Further, the addition of tranexamic acid (a lysine analogue, which results in a reduction in the activation and action of plasmin), reduced AAA formation and abolished AAA rupture in the TAFI-/- [19], implying that it was the effects of plasmin that resulted in the formation and rupture of AAA. It may be that the important pathological processes in the formation of experimental AAA differ between the models, with inflammation following thrombus formation being a more key factor in the breakdown of the aortic wall, and thus AAA formation, in the Ang II model compared with the elastase model. Indeed, in a previous study, when Ang II was infused into OPN-/-ApoE-/- mice, they were protected from AAA formation [25], again implying an important role for inflammation in this particular model of AAA.

Given the positive effects of the inhibition of plasmin-mediated TAFI activation on the formation of AAA *in-vivo*, we next studied if this could affect the progression of AAA in this model. To do this, mice were administered with a dose of MA-TCK26D6 (or saline control) one week after the initiation of the Ang II infusion, once AAA had already been established. As, based on our own pilot data, not all ApoE-/- mice treated with Ang II were expected to develop AAA, mice underwent abdominal ultrasound scans prior to any injection in order to be able to measure the pre-treatment AAA size. There was no mortality following either injection. There was no effect of plasmin-mediated TAFI inhibition on AAA progression in this model; the change in AAA size was comparable between the treatment group and the controls. There was a reduction in the distensibility of the aorta, as would be expected with AAA formation, in both groups, and again, there was no effect on the change in aortic distensibility with MA-TCK26D6 treatment. Together, these data show that later in the Ang II model of AAA, inhibition of TAFI does not have any effect on AAA development. It is likely that after one week, the pathological processes at work in this model are already too well established to be influenced by the inhibition of TAFI activation. This further supports our hypothesis that, in the Ang II model of AAA, it is the combination of initial intra-mural thrombus formation and an inflammatory response that drives AAA formation. Once thrombus is formed, and the inflammatory response has been initiated, the addition of an inhibitor that prevents further thrombus formation has no effect on outcome.

Given that late treatment with MA-TCK26D6 did not affect AAA progression, it seems unlikely that these data will be directly transferable to human disease, as treatment for AAA can only be initiated following diagnosis, once the disease process is already underway. However, our data demonstrate that early inhibition of plasmin-mediated TAFI activation confers a survival advantage and reduces AAA formation in an Ang II model of AAA. Further studies are required to investigate the role of thrombosis, fibrinolysis and subsequent inflammatory response in the early stages of AAA.

## Supporting information

S1 Fig Fibrin clot structure in the Angiotensin II model of AAA. Turbidity and turbidity lysis was performed using plasma from mice with AAA (n = 7) and mice treated with saline control (n = 8). There was no difference in the time taken to reach the maximum OD (11.11±2.57 mins vs. 13.46±2.32 mins, panel A), the maximum OD (0.10±0.07 vs. 0.09±0.02, panel B) or the time to half lysis (50.59±23.31 mins vs. 41.21±14.93 mins, panel C). Fibrin clots were formed which incorporated FITC-labelled fibrinogen, in order to allow for the microscopic study of the fibrin clot structure. This confirmed that there was no difference in the fibrin fibre density in mice treated with Ang II developing AAA and controls (286.3±260.4 fibres/100μm^2^ vs. 361.3±217.2 fibres/100μm^2^, panel D). Sample images of the fibrin clot structure are shown in Panel E.
(TIF)
