# Supplementary figures and images for: Inhibition of plasmin-mediated TAFI activation may affect development but not progression of abdominal aortic aneurysms
Source: PLoS One. 2017 May 4;12(5):e0177117. doi: 10.1371/journal.pone.0177117 (PMC5417566; doi:10.1371/journal.pone.0177117)

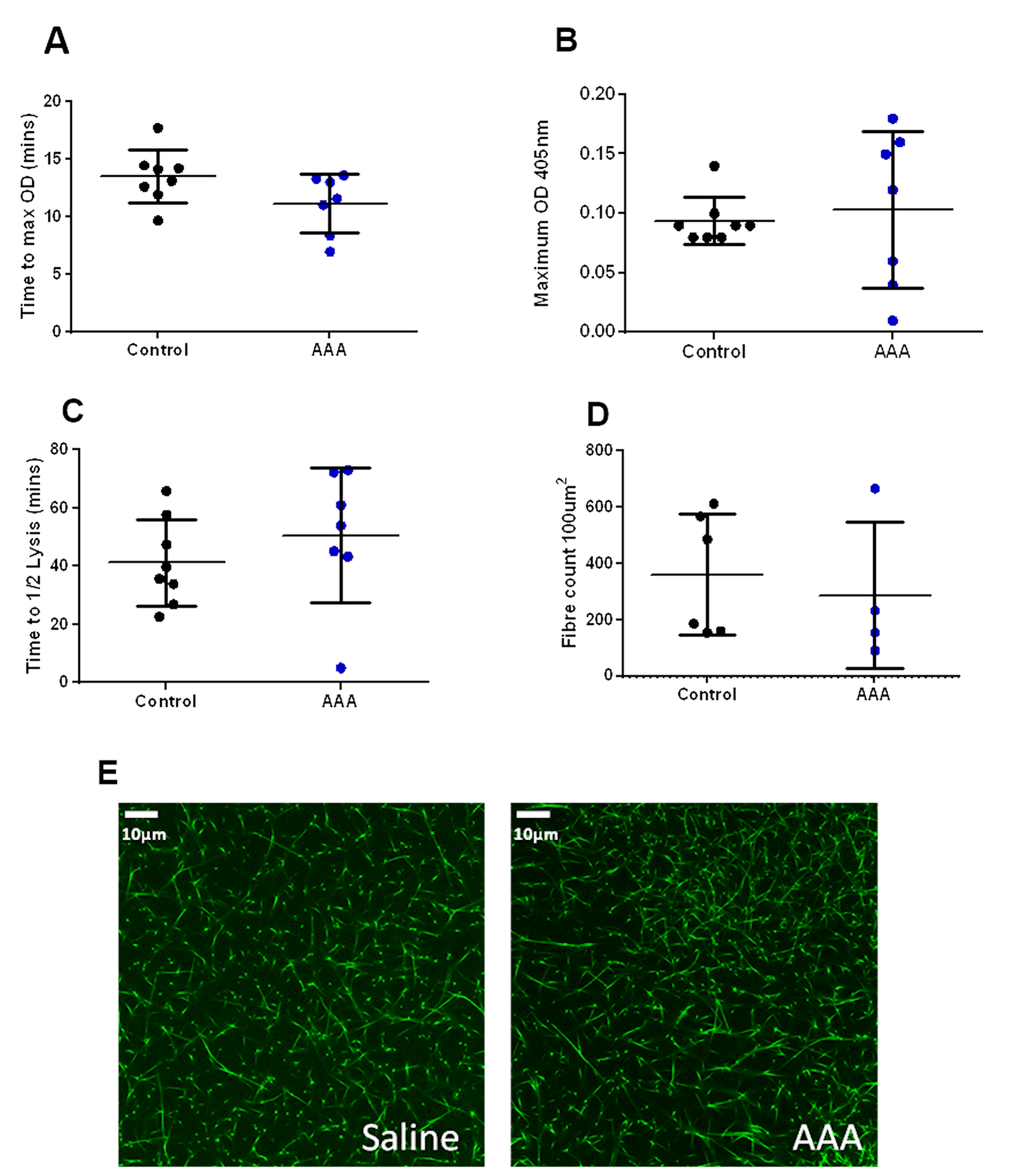

Supplement: S1 Fig — Turbidity and turbidity lysis was performed using plasma from mice with AAA (n = 7) and mice treated with saline control (n = 8). There was no difference in the time taken to reach the maximum OD (11.11±2.57 mins vs. 13.46±2.32 mins, panel A), the maximum OD (0.10±0.07 vs. 0.09±0.02, panel B) or the time to half lysis (50.59±23.31 mins vs. 41.21±14.93 mins, panel C). Fibrin clots were formed which incorporated FITC-labelled fibrinogen, in order to allow for the microscopic study of the fibrin clot structure. This confirmed that there was no difference in the fibrin fibre density in mice treated with Ang II developing AAA and controls (286.3±260.4 fibres/100μm2 vs. 361.3±217.2 fibres/100μm2, panel D). Sample images of the fibrin clot structure are shown in Panel E. (TIF) [file pone.0177117.s001.tif]
